# COMPARING GAIT AND HIP SCORES IN FEMORAL NECK AND INTERTROCHANTERIC FRACTURES

**DOI:** 10.1590/1413-785220233102e261336

**Published:** 2023-06-09

**Authors:** SEFA AKTI, HAKAN ZEYBEK

**Affiliations:** 1. Cumhuriyet University, Department of Orthopaedics and Traumatology, Sivas, Turkey.; 2. İzmir Katip Celebi University, Ataturk Training and Research Hospital, Department of Orthopaedics and Traumatology, İzmir, Turkey.

**Keywords:** Smartphone, Gait analysis, Hip fractures, Hemiarthroplasty, Smartphone, Análise da marcha, Fraturas do quadril, Hemiartroplastia

## Abstract

**Objective:**

Treatment modality is controversial in the unstable IT fractures. Ideal hemiarthroplasty treatment for unstable IT fractures should be comparable to that for FN fractures. Therefore, the aim of this study was to compare patients who underwent cementless hemiarthroplasty for a diagnosis of FN and unstable IT in terms of clinical outcomes, functional scores, and smartphone-based gait analysis data.

**Methods:**

Case matching was applied to 50 patients with FN fracture and 133 patients with IT fracture who underwent hemiarthroplasty treatment, they were compared in terms of, preoperative and postoperative walking status, and Harris hip scores. Smartphone-based gait analysis was applied to 12 patients in the IT group and 14 patients in the FN group who could walk without support.

**Results:**

There was no significant difference between patients with IT and FN fractures regarding Harris hip scores, preoperative, and postoperative walking status. In the gait analysis, gait velocity, cadence, step time, step length, and step time symmetry values were observed to be significantly better in patients in the FN group.

**Conclusion:**

Cementless hemiarthroplasty operations for unstable IT fractures have similar hip scores to FN fractures. However, the walking speed and walking symmetry data were seen to be worse. This result should be considered in the selection of appropriate treatment. Level of evidence III; Retrospective study.

## RESUMO

**Objetivo:**

O tratamento das fraturas instáveis da IT possui modalidades de tratamento com diferentes teorias. Hemiartroplastia é o tratamento ideal para fraturas instáveis (IT), devendo ser comparável à hemiartroplastia para fraturas do colo femoral (FN). Portanto, o objetivo deste estudo foi comparar pacientes que foram submetidos a hemiartroplastia não cimentada para diagnóstico do FN e IT instável em seus resultados clínicos, considerando a escala de estado funcional e a análise dos dados de habilidade de marcha coletadas por um smartphone.

**Métodos:**

A combinação de casos foi aplicada a 50 pacientes com fratura FN e 133 pacientes com fratura IT submetidos ao tratamento de hemiartroplastia, a habilidade de marcha pré e pós-operatório, incluindo suas pontuações Harris Hip, foram comparadas. A análise de marcha foi executada com smartphone em 12 pacientes do grupo IT e 14 pacientes do grupo CF, que conseguiam andar sem apoio.

**Resultados:**

Não foram encontradas diferenças significativas entre os pacientes com fraturas IT e FN em relação às pontuações Harris Hip nem quanto ao estado de marcha pré e pós-operatório. Na análise da marcha, os valores de velocidade, cadência, tempo de passo, comprimento do passo e simetria do tempo de passo foram significativamente melhores nos pacientes do grupo FN.

**Conclusão:**

As operações de hemiartroplastia não cimentada para fraturas instáveis de IT, têm pontuação de quadril semelhantes às fraturas FN. Entretanto, os dados de velocidade de caminhada e simetria de caminhada mostraram-se inferiores. Esses resultados devem ser considerados na escolha do tratamento adequado.Nível de evidência III; Estudo retrospectivo.

## INTRODUCTION

The incidence of proximal femur fractures in the elderly population is increasing around the world.^1 , 2^ As these fractures constitute a global health problem with high morbidity and mortality rates, appropriate treatment has become more important.^3^ In the treatment of displaced femoral neck (FN) fractures in elderly patients, hemiarthroplasty is a globally accepted and widely used method.^4^

However, the optimal treatment for intertrochanteric femur (IT) fractures is controversial. Intramedullary nailing is a frequently preferred method because of the high potential for union due to the rich vascular network of the intertrochanteric region, the biomechanical advantages, and that it can be performed with a minimally invasive surgical technique.^5 - 7^ Despite the advantages of intramedullary nailing, it is one of the most preferred methods in the treatment of hemiarthroplasty, sometimes due to physician and sometimes patient-related factors.

**Figure 1:**
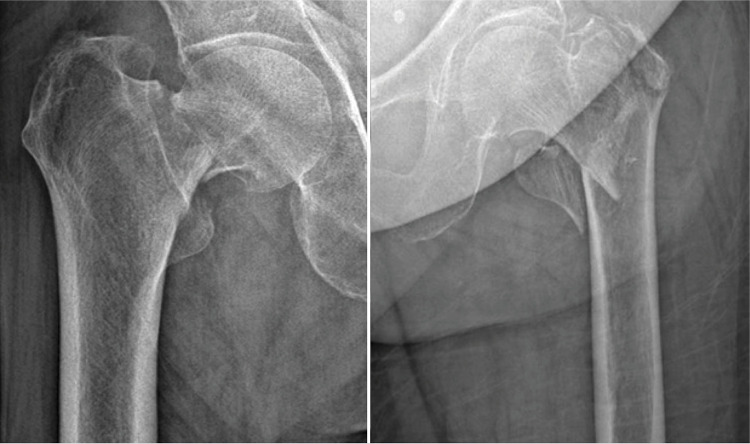
Radiographs of a 78-year-old male patient with a displaced femoral neck fracture and an 83-year-old female patient with an unstable intertrochanteric fracture.

Therefore, various hemiarthroplasty methods have been reported in the treatment of unstable IT fractures, aiming to prevent complications mentioned above and allow full weight-bearing immediately after surgery.^8 , 9^ Unlike FN fractures, the common purpose of these methods is to maintain the stability of the prosthesis despite the impaired abductor mechanism that occurs in IT fractures. ( Figure 1 ) Therefore, ideal hemiarthroplasty treatment for unstable IT fractures should be comparable to hemiarthroplasty treatment for FN fractures. However, there are few studies that have compared the results of hemiarthroplasty for FN fractures with hemiarthroplasty for unstable IT fractures.^10 , 11^ The hypothesis of this study was that hemiarthroplasty operations performed for IT region fractures would yield clinical and functional results similar to those of hemiarthroplasty operations performed for FN fractures, with an average follow-up of more than 1 year. Therefore, the aim of the study was to compare the clinical results, functional scores and smartphone-based gait analysis data of patients who underwent hemiarthroplasty for FN and IT fractures.

## MATERIAL AND METHODS

**Figure 2:**
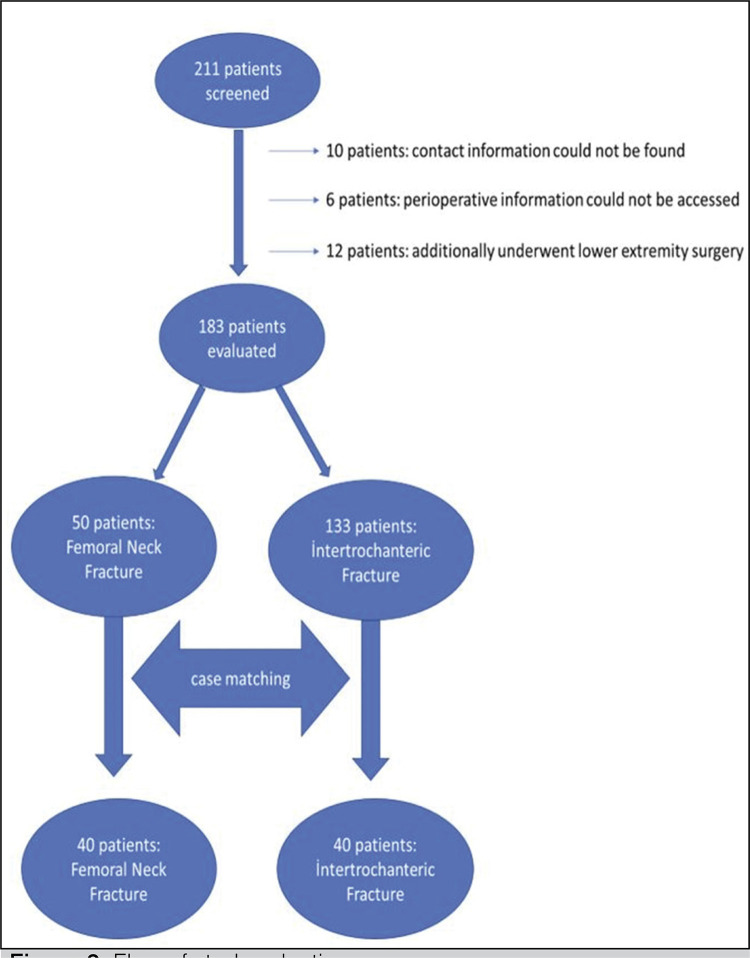
Flow of study selection.

Approval for the study was granted by the Local Ethics Committee (Ethical approval: File Number: 2100005070). All involved subjects gave informed consent to the work. The hospital archives were screened to identify patients aged ≥70 years who underwent cementless hemiarthroplasty with proximal femur fracture between 2014 and 2020. A total of 211 patients were identified, of which 28 were excluded for various reasons; 10 could not be contacted, perioperative information was not available for 6, and 12 patients had another lower extremity operation. Thus, a total of 183 patients were evaluated, comprising 50 patients with displaced FN fracture and 133 patients with unstable IT fracture. Case matching was applied to 50 FN patients and 133 IT fracture patients using the factors of age, gender, body mass index (BMI), bone mineral density (BMD), American Society of Anesthesiologists (ASA) score, and pre-injury walking status. As a result of the matching process, 40 patients were in the IT group and 40 patients were in the FN group as the control group. ( Figure 2 ) The demographic data of the patients before and after matching are presented in Table 1 .

**Table 1:** Patients included in the study and statistical matching between the two groups.

	Patients included in the study			After matching two groups.		
Case variables	Intertrochanteric group (n=133)	Femoral neck group (n= 50)	P value	Intertrochanteric group (n=40)	Femoral neck group (n=40)	P value
Age (years)	83.11±6,32	82.72 ±7,62	0,724	82.48± 6.714 (71-95)	82.53± 7.383 (71-98)	0.975
Gender			0.414			1.000
Male	54	17		14	14	
Female	79	33		26	26	
Affected side			0.160			0.502
Right	59	28		19	22	
Left	74	22		21	18	
Bone mineral density (T-score)	3.759±0.52	3.808±0.46	0.569	3.785±0.49	3.81±0.48	0.786
Body mass index(kg/m^2^)	26.58±3.48	26.40±2.62	0.742	26.33±3.02	26.58±2.64	0.695
ASA score	2.53±0.646	2.42±0.642	0.322	2.40±0.632	2.33±0.572	0.580
Pre-injury walking ability			0.970			0.695
Without support	75	28		25	23	
With support	50	19		13	16	
Unable to walk	8	3		2	1	
Anesthesia			0.607			0.556
General:regional	11:122	3:47		2:38	1:39	
Follow-up period(months)	17.80±12.68 (6-72)	15.26±11.48 (6-75)	0.218	18.58±12.03 (6-66)	15.03±12.45 (6-75)	0.198

ASA: American Society of Anesthesiologists. Values presented: mean±standard deviation.

All operations were performed using a standard posterolateral approach. For patients with FN fracture, a cementless Biomet^®^ Bi-Metric Plasma Spray porous coating tapered femoral stem was used with the standard surgical technique. In patients with IT fracture, a TipMed^®^ S-2 anatomical modular hip prosthesis-uncemented, distal handle femoral stem and perforated stem combination were used. Major and minor trochanter fractures and short external rotator muscle groups were fixed with cerclage wire passed through the perforations. The short external rotator muscle groups and capsule were sutured to the posterior border of the gluteus medius in all patients. In all patients, length was obtained by measuring the contralateral leg. A bipolar head was used in all patients.

Antibiotic prophylaxis with cefazolin was administered to the patients 30-60 minutes before the operation and was continued for up to 48 hours postoperatively. Low molecular weight heparin was administered daily subcutaneously and continued for three weeks. Weight-bearing was permitted on the operated extremity on the first postoperative day and patients were advised to use a walker until they had regained sufficient muscle strength and balance. Excessive flexion and adduction were not permitted for six weeks postoperatively.

The patients were followed up with clinical and radiological examinations at 6 weeks, 6 months, 1 year and annually thereafter. The Harris hip score (HHS)^12^ was recorded. The pre-injury walking ability of the patients was evaluated in three categories as without support, with support, and unable to walk. These 3 categories were also used for the postoperative evaluations of walking ability.

**Figure 3:**
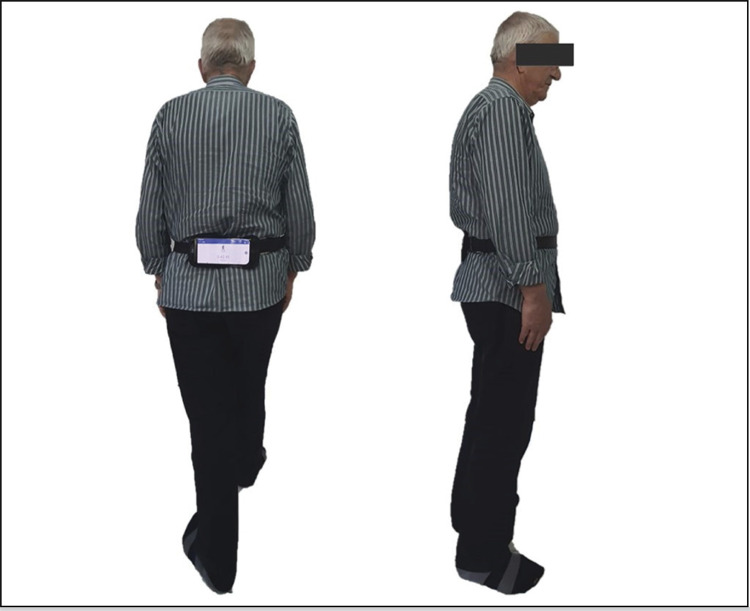
Back and side view of smartphone placed on the body.

At the final follow-up examination, smartphone-based gait analysis was applied to patients from both groups who could walk without support. These patients, who could walk continuously without the assistance of another person or a walking aid, walked barefoot along a 10-m walkway at a self-selected walking speed with a smartphone attached by a belt to the body above the third lumbar vertebrae in a horizontal orientation. ( Figure 3 )^13 - 15^

**Figure 4:**
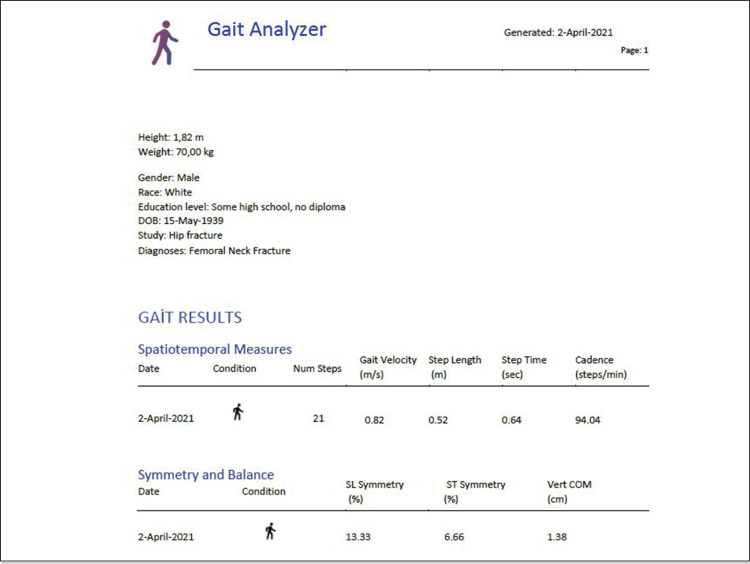
Output from the application for a femoral neck fracture patient at 18 months after surgery.

The gait analysis was applied using the Gait Analyzer version 1.0.1 (Control One LLC, NM, USA) smartphone application running on Samsung Galaxy Note 10 Plus smartphone (143.3×71.1×6.3 mm; 141 g), as described in a previous study.^15^ Calibration was performed by walking 5 meters before testing each new patient. The gait data during calibration were not included in the assessment. The data collected by the Acceleration Sensor LSM6DSO (STMicroelectronics, Geneva, Switzerland) were low-pass filtered before further analysis (fourth-order zero-lag Butterworth filter at 20 Hz). In the new graphic, the heel strike time points were determined using the relevant mathematical formulas. The gait velocity, step time (ST), step length (SL), cadence, step length symmetry, step time symmetry, and vertical COM (vert-COM) parameters were measured in all patients. ( Figure 4 )

### Statistical analysis

Statistical analysis of the data obtained in the study was performed using SPSS version 22 software (SPSS Inc., Chicago, IL, USA). The characteristics of the patient populations were analyzed using the Chi-square test for categorical variables and the student’s t-test for continuous variables. The Mann-Whitney U test was applied to data that did not show normal distribution. A value of p<0.05 was accepted as statistically significant in all the analyses.

## RESULTS

No significant difference was observed between the assisted walking times of the patients. No significant difference was observed between the post-operative walking ability and final Harris Hip scores of the 33 IT patients and 36 FN patients without mortality (p>0.05). ( Table 2 ) Smartphone-based gait analysis was applied to 14 patients in the FN group and 12 patients in the IT group who were able to walk without support. The gait velocity, cadence, step time, step length and step time symmetry values were observed to be significantly better in the FN group patients (p<0.05). No significant difference was determined between the groups of patients applied with gait analysis in respect of age, gender, BMI, side, leg length, step length symmetry, and vertical COM values (p>0.05). ( Table 3 )

**Table 2:** Postoperative ambulatory capability and Harris Hip scores of the two groups.

Variables	Intertrochanteric group (n = 33)	Femoral neck group (n = 36)	P-value
Starting day of ambulation with support	4.83±3.1	4.65±2.42	0.779
Final Walking Ability			0.894
Without support	12	14	
With support	19	19	
Unable to walk	2	3	
Total number of patients	33	36	
Harris Hip score	65.58±9.04	64.33±10.34	0.598
Number of patients	33	36	

Values presented: mean±standard deviation.

**Table 3:** Gait analyses of the patients able to walk without support.

Case variables	Intertrochanteric group (n=12)	Femoral neck group (n=14)	P value
Age (years)	77.33±3.47	76±03	0.307
Gender			0.356
Male	1	3	
Female	11	11	
BMI	26.5±3.06	27.0±2.14	0.630
Affected side Right:left	7:5	5:9	0.249
Right leg length	87.42±8.68	89.36±8.96	0.582
Left leg length	87.58±8.55	89.14±8.5	0.647
Gait velocity	0.59±0.12	0.74±0.07	0.001
Cadence	75.45±14.13	86.72±8.48	0.019
Step time	0.82±0.19	0.69±0.08	0.031
Step length	0.46±0.058	0.50±0.05	0.044
Step time symmetry	30.05±31.04	11.90±17.40	0.002
Step length symmetry	26.68±13.71	20.16±8.69	0.154
Vertical COM	1.43±0.34	1.52±0.31	0.485

Values presented: mean±standard deviation.

## DISCUSSION

The most important finding of this study was that the gait velocity value of the patients who underwent arthroplasty due to FN was significantly better than that of the patients who underwent arthroplasty due to IT. There are many studies showings that life expectancy increases with increasing gait velocity in elderly patients.^16^ However, in the current study, no significant difference was observed between the FN and IT patients in respect of mortality rates, which was consistent with the findings of the few similar studies in literature.^10^

The selection of the femoral stem can be difficult for the most appropriate treatment of IT fractures. Many of the studies on this subject have made comparisons between patients with different bone strength, muscle strength and, more importantly, fracture morphology. Taking the treatment results of FN fractures, in which the intertrochanteric region muscle and bone structures remain intact, as a reference, can bring another perspective to measuring the effectiveness of the treatment of IT fractures and reaching the ideal treatment option. In a study by Chang et al., patients applied with cementless prosthesis for FN and IT fractures were compared.^10^ No significant difference was determined in respect of the amount of bleeding, blood transfusion and HHS. Similar results were observed in the current study. However, the functional status of patients with PROMs may not always be parallel.^17^ It has been shown that while postoperative HHS values improved in patients with unstable IT fractures, the gait parameters seen in gait analysis did not improve at the same rate.^17^

Various applications were developed and installed on smartphones, and with these applications, gait analysis became possible without the need for an additional program, computer or engineering knowledge. Several recent articles have been published showing that smartphone-based gait analysis is reliable and valid and therefore smartphone-based gait analysis was used in this study.^13 , 14 , 18^

The normal function of the muscles attached to the proximal femur is very important for both prosthesis stability and walking functions.^19^ Dysfunction in these muscles in IT fractures is expected to impair the gait parameters measured in patients. In a study of unilateral partial hip arthroplasty patients, the average walking speed was found to be 0.5 m/s.^11^ In another gait analysis study of patients with FN and IT hip fractures, it was stated that walking speed and gait symmetry parameters observed in IT region fractures were worse.^20^ These findings seem to be compatible with those of the current study. In addition, the mean walking speed in patients with hip fracture has been reported to be 0.6 m/s (SD=0.2). In the current study, the mean walking speed was found to be 0.67 m/s (SD=0.12) in all patients with FN and IT fractures. Although gait symmetry parameters are the main parameters of gait analysis, it has been shown that improving these parameters reduces energy consumption in patients.^21^ The main symmetry parameters in spatiotemporal gait analysis are step time symmetry and step length symmetry parameters. However, the relationship between these two parameters and their contribution to energy consumption is a matter of debate.^22 , 23^ In a study in which healthy individuals walked on a treadmill at increasing speeds, it was shown that step time symmetry values deteriorated while step length symmetry values remained constant at varying speeds. The authors stated that the step time symmetry value was broken first so that the body can aim to keep the step length symmetry value constant to ensure optimal energy consumption.^24^ These findings are consistent with those of the current study. While no significant difference was observed in the step length symmetry value between the current study groups, a significant difference was determined in the step time symmetry value.

The strength of this study was that it was a case-control study conducted in a single centre. However, the study had some limitations such as being retrospective, having a relatively small sample size, the lack of HHS values during follow-up, the evaluation of only final values, and that gait parameters could not be evaluated in assisted walking patients.

## CONCLUSION

Hemiarthroplasty operations in hip region fractures should not be considered as a single type of an operation due to their results. The same choice produces different results depending on the region of fracture. The orthopaedic surgeon should consider that the hemiarthroplasty treatment for IT fractures will have worse gait results compared to the hemiarthroplasty treatment for FN fractures. Hemiarthroplasty treatment should not be preferred as much as possible in IT fractures.
